# Can Serum Nitrosoproteome Predict Longevity of Aged Women?

**DOI:** 10.3390/ijms21239009

**Published:** 2020-11-27

**Authors:** Daniele Capitanio, Pietro Barbacini, Beatrice Arosio, Franca Rosa Guerini, Enrica Torretta, Fabio Trecate, Matteo Cesari, Daniela Mari, Mario Clerici, Cecilia Gelfi

**Affiliations:** 1 Department of Biomedical Sciences for Health, University of Milan, 20090 Segrate (MI), Italy; daniele.capitanio@unimi.it (D.C.); pietro.barbacini@unimi.it (P.B.); 2 Geriatric Unit, Fondazione IRCCS Ca’ Granda Ospedale Maggiore Policlinico, 20122 Milan, Italy; beatrice.arosio@unimi.it; 3 Department of Clinical Sciences and Community Health, University of Milan, 20122 Milan, Italy; matteo.cesari@unimi.it; 4 IRCCS Fondazione Don Carlo Gnocchi ONLUS, 20148 Milan, Italy; fguerini@dongnocchi.it (F.R.G.); ftrecate@dongnocchi.it (F.T.); mario.clerici@unimi.it (M.C.); 5 IRCCS Istituto Ortopedico Galeazzi, 20161 Milan, Italy; enrica.torretta@grupposandonato.it; 6 Geriatric Unit, IRCCS Istituti Clinici Scientifici Maugeri, 20138 Milan, Italy; 7 Laboratorio Sperimentale di Ricerche di Neuroendocrinologia Geriatrica ed Oncologica, IRCCS Istituto Auxologico Italiano, 20145 Milan, Italy; daniela.mari@unimi.it; 8 Department of Pathophysiology and Transplantation, University of Milan, 20122 Milan, Italy

**Keywords:** proteomics, nitrosative stress, aging, cardiovascular disease, muscle atrophy

## Abstract

Aging is characterized by increase in reactive oxygen (ROS) and nitrogen (RNS) species, key factors of cardiac failure and disuse-induced muscle atrophy. This study focused on serum nitroproteome as a trait of longevity by adopting two complementary gel-based techniques: two-dimensional differential in gel electrophoresis (2-D DIGE) and Nitro-DIGE coupled with mass spectrometry of albumin-depleted serum of aged (A, *n* = 15) and centenarian (C, *n* = 15) versus young females (Y, *n* = 15). Results indicate spots differently expressed in A and C compared to Y and spots changed in A vs. C. Nitro-DIGE revealed nitrosated protein spots in A and C compared to Y and spots changed in A vs. C only (*p*-value < 0.01). Nitro-proteoforms of alpha-1-antitripsin (SERPINA1), alpha-1-antichimotripsin (SERPINA3), ceruloplasmin (CP), 13 proteoforms of haptoglobin (HP), and inactive glycosyltransferase 25 family member 3 (CERCAM) increased in A vs. Y and C. Conversely, nitrosation levels decreased in C vs. Y and A, for immunoglobulin light chain 1 (IGLC1), serotransferrin (TF), transthyretin (TTR), and vitamin D-binding protein (VDBP). Immunoblottings of alcohol dehydrogenase 5/S-nitrosoglutathione reductase (ADH5/GSNOR) and thioredoxin reductase 1 (TRXR1) indicated lower levels of ADH5 in A vs. Y and C, whereas TRXR1 decreased in A and C in comparison to Y. In conclusion, the study identified putative markers in C of healthy aging and high levels of ADH5/GSNOR that can sustain the denitrosylase activity, promoting longevity.

## 1. Introduction

Longevity is the result of multiple factors including genetic, epigenetic, and lifestyle; however, circulating molecules able to predict the evolution from healthy toward stressor events overwhelming the antioxidant capacity of the entire organism are still absent. Aging is characterized by a progressive decline of the muscle functional performance (i.e., sarcopenia), which contributes to frailty, defined as the vulnerability of the body to exogenous and endogenous stressors [1]. One of the theories proposed to explain the aging process is based on the free radical theory proposed by Denhan Harman in 1956, suggesting that accumulation of oxygen species overcomes the cellular anti-oxidative capacity [2]. Following this theory, post-translational modifications of proteins such as nitrosation and peroxidation can be associated with the aging process including sarcopenia.

Recent results indicate that the increase in reactive oxygen (ROS) and nitrogen (RNS) species are key factors of cardiac and vasculature homeostasis and disuse-induced muscle atrophy [3,4]. The effects and the molecular mechanisms that regulate oxidative stress, in particular those related to nitrosative stress and the role of antioxidant defense in human skeletal muscle and at the whole-body level, are still poorly understood. Nitric oxide (NO) is an important signaling molecule produced endogenously from NO synthase (NOS). NO acts on proteins by means of selective covalent modification of cysteine residues through a redox reaction to form nitroso thiols (SNOs) [5]. This mechanism regulates a wide range of cellular functions and signal processes such as neurotransmission, muscle relaxation, and apoptosis. Physiologically, NO in cells is maintained at a minimum level to allow the carrying out of its regulatory role in various signal pathways. Conversely, the excessive production of NO triggers nitrosative stress and induces changes in S-nitrosated protein levels. It has been described that a prolonged muscle disuse, such as that observed in bedridden subjects, compromises the Ca^2+^ homeostasis that increases muscle deficit with alteration of its functional capabilities. This dysregulation can be improved with exercise as a countermeasure [6,7,8]. On muscle, the ryanodine receptor 1 (RYR1) nitrosation is directly related to the loss of muscle mass and function [9,10], and its regulation is a critical condition for the muscular homeostasis maintenance. In cardiac tissue, nitrosation of the ryanodine receptor 2 (RYR2) regulates peripheral vascular tone and β-adrenergic agonist-stimulated cardiac contractility [11]. It has also been demonstrated that the denitrosylase alcohol dehydrogenase 5/S-nitrosoglutathione reductase (alcohol dehydrogenase 5/S-nitrosoglutathione reductase (ADH5/GSNOR)) is downregulated in cells and animal models of aging and that mRNA levels from circulating peripheral blood mononuclear cells (PBMC) are retained in centenarians [12,13]. We expect that nitrosation of proteins could be a more general event and we assume that markers of nitrosative stress can be found not only in cardiac and skeletal muscle tissues but also in bodily fluids.

In this context, we would like to understand if there is a relationship between circulating biomarkers and loss of muscle mass and function or if other mechanisms contribute to longevity. Furthermore, it has been recently demonstrated that biomarkers of successful aging are sex-related [14,15], and thus gender has to be considered. In a previous study conducted in male aged subjects, the loss of muscle function was connected to the loss of phosphorylatable myosin light chain, which regulates the velocity of fiber contraction, and of changes in muscle metabolism, resulting in fat accumulation [16,17]. In astronauts undergoing 6 months of space flight, characterized by a loss of muscle mass and function at landing, deregulated miRNA were associated with microgravity exposure, pointing to a strong stress response caused by space flight that involved muscle tissue and proinflammatory molecules. In particular, the muscle biopsy of one of the astronauts showed a 80% increase of alpha-1-antitrypsin, an acute phase protein, and of its target miR-126-3p, suggesting that serum can tell us something about the muscle function decline and the effect at the whole body-level of a stressful condition such as a prolonged space flight [18]. More recently, sarcopenia in men was found to be associated with high cholesterol and lower creatinine levels. Conversely, in women, the maintenance of normal gait speed was found to be associated with a lower percentage of body fat and lower lactate dehydrogenase levels [15].

Several important studies have recently addressed the issue of a differential protein expression associated with aging, identifying a set of increased proteins as putative biomarkers of aging [19,20,21,22]. However, these studies on circulating proteome did not suggest any mechanism that can be targeted to counteract the aging decline affecting the entire organism.

In an attempt to contribute, we addressed the issue of nitrosignalling following levels of nitrosated proteins in serum of aged individuals and centenarians to find molecules that can be targeted to promote the evolution toward longevity and, indirectly, to expand the healthy age interval that have allowed centenarians to reach their exceptional age. It has been recently described that S-nitrosation increases the rate of mitochondrial fragmentation and inhibits mitophagy, which is tightly associated with the aging phenotype and decline of muscle mass and function [23]. The study indicates that excessive S-nitrosation of key mitochondrial proteins contributes to aging and related disorders [24,25,26,27], and this process is regulated by denitrosylases such as thioredoxin/thoredoxin reductase (Trx/TrxR) and ADH5/GSNOR [28,29]. Thioredoxin reductase, together with the reduced form of nicotinamide adenine dinucleotide phosphate (NADPH) and thioredoxin, is a component of the thioredoxin system that, with the glutathione–glutaredoxin system, controls the redox environment of mammalian cells. The Trx system protects cells from oxidative and nitrosative stress through its disulfide reductase activity regulating protein dithiol/disulfide balance. ADH5/GSNOR regulates intracellular concentration of reactive NO by catalyzing the breakdown of S-nitrosoglutathione (GSNO), a NO donor for cysteine thiols, thus indirectly modulating SNO formation through GSNO-mediated protein S-nitrosation. This suggests that these two systems target different molecules, since GSNOR specifically denitrosates GSNO, whereas the cytoplasmic and mitochondrial thioredoxins mediate denitrosation of multiple S-nitrosated proteins in relation to stimulus, substrate, and localization [30].

In an attempt to find modified molecules involved into the aging process and in the loss of muscle mass characterizing aging, we adopted a two-dimensional difference in gel electrophoresis (2-D DIGE) gel-based analysis of the serum proteome in conjunction with Nitro-DIGE and mass spectrometry of aged (A) women versus young (Y) women and centenarians (C) in order to identify putative markers associated with aging.

## 2. Results

### 2.1. Assessement of Muscle Performances

**Figure 1:**
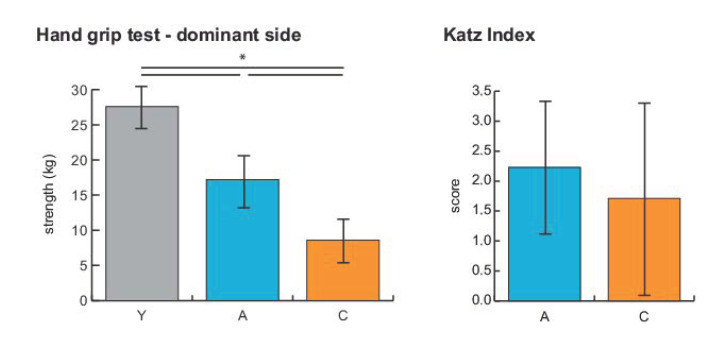
Histograms showing the mean (± SD) maximum hand grip strength (kg) and the Katz Index scores observed among the young (Y), aged (A), and centenarian (C) female subjects enrolled in this study (* = significant difference, ANOVA and Tukey, *p*-value < 0.05).

The loss of muscle force and function associated with sarcopenia was determined by hand grip test and short physical performance battery (SPPB) in aged women, and by hand grip test in centenarian women. SPPB indicated in five subjects a score of 7 or higher, while for the remaining subjects, the values ranged from 0 to 3, indicating a functional decline, as shown in Table S1. Figure 1 shows the muscle force decline determined by hand grip test in aged and centenarian women. The mean maximum hand grip strength observed among young subjects was 27.6 kg, whereas it decreased to 17.2 kg in aged women and to 8.6 kg in centenarians. The decline was increased in centenarians, although four subjects had a hand grip test value comparable with some of the aged women, and only three of them (aged 106, 107, and 113 years old, respectively) showed a significant decline of force levels (below 10 kg). Nonetheless, these three subjects had body mass index (BMI) values comparable to subjects of the same cohort. The Katz index of independence in activities of daily living did not show significant variations (Student’s *t*-test, *p* < 0.05) among aged and centenarians, due to the great variability in both groups (2.2 ± 1.1 and 1.7 ± 1.7 for aged women and centenarians, respectively). Furthermore, 12 out of 15 aged subjects and 11 out of 15 centenarians had hypertension and were pharmacologically treated.

### 2.2. 2-D DIGE and Nitro-DIGE Proteomics

**Figure 2:**
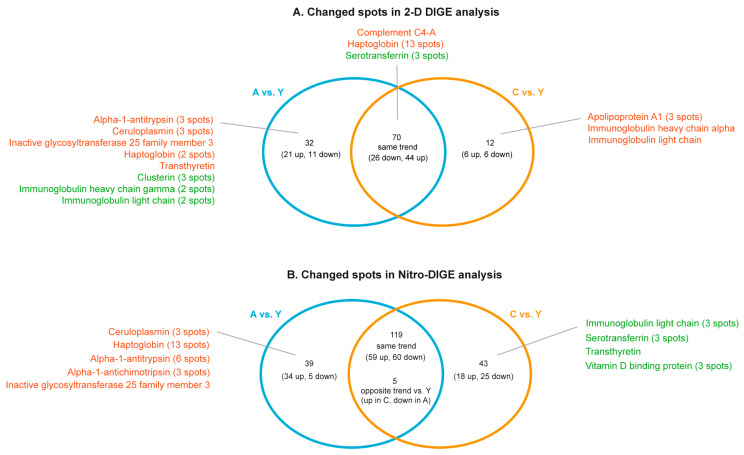
Schematic diagrams resuming findings obtained from two-dimensional differential in gel electrophoresis (2-D DIGE) (**A**) and Nitro-DIGE (**B**) proteomic analyses conducted on albumin-depleted sera from young (Y), aged (A), and centenarian (C) women. Protein names and number of identified spots (corresponding to different proteoforms) were reported (red = increased; green = decreased levels in A and/or C vs. Y, ANOVA and Tukey, *n* = 6 (2-D DIGE) or *n* = 9 (Nitro-DIGE), *p*-value < 0.01).

Albumin-depleted serum samples were analyzed by 2-D DIGE and Nitro-DIGE to evaluate changes in protein abundance and in their protein S-nitrosation levels among A, C, and Y subjects. Overall, 2-D DIGE revealed 120 changed over 978 matched spots among all gels. Among them, 114 spots were differently expressed (ANOVA and Tukey, *n* = 6, *p*-value < 0.01) in A and C compared to Y subjects (32 significantly changed in A, 12 in C, 70 changed with the same trend both in A and C vs. Y) (Figure 2A). Identification data are shown in Tables S2 and S3, and in Figure S1. Nitro-DIGE analysis revealed 206 nitrated protein spots as changed (ANOVA and Tukey, *n* = 9, *p*-value < 0.01) in A and C compared to Y (39 changed in A, 43 in C, whereas 119 changed with the same trend both in A and C vs. Y, and 5 counter-regulated in A vs. Y compared to C vs. Y) (Figure 2B).

**Figure 3:**
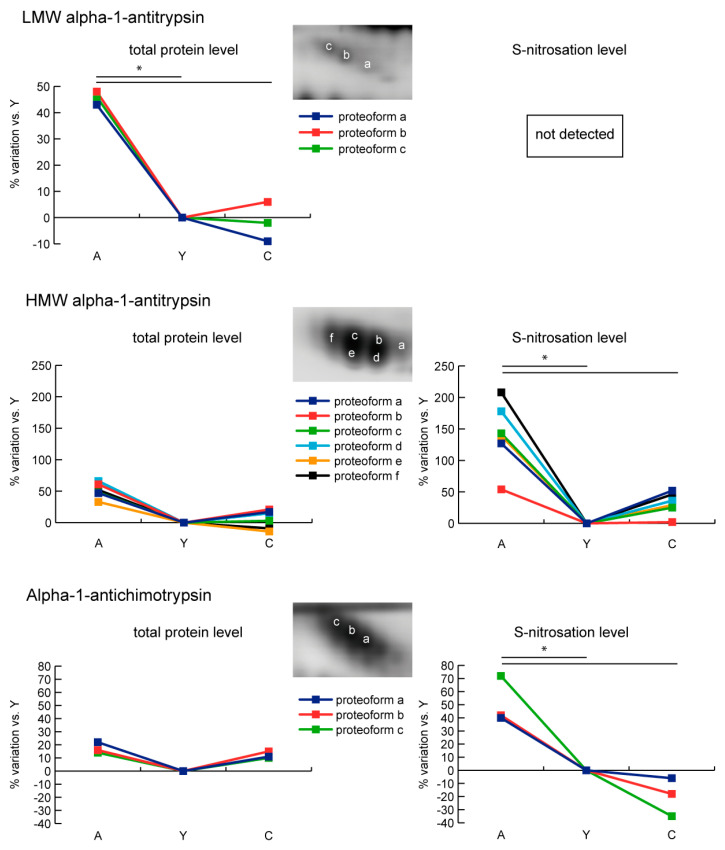
Line charts illustrating alpha-1-antitripsin (SERPINA1) and alpha-1-antichimotripsin (SERPINA3) proteoform abundance and S-nitrosation level variations (%) in sera of aged (A) and centenarian (C) compared to young (Y) women. Proteins were divided according to the statistical significance of the test (* = significant difference, ANOVA and Tukey, *n* = 6 (2-D DIGE) or *n* = 9 (Nitro-DIGE), *p*-value < 0.01).

Mass spectrometry analysis allowed the identification of alpha-1-antitripsin (SERPINA1). SERPINA1 is composed by high (HMW) and low molecular weight (LMW) proteoforms. Three LMW spots were increased in A only and were not S-nitrosated. By contrast, six HMW spots were unchanged and their level of S-nitrosation increased in A but not in C. Another identified protein was alpha-1-antichimotripsin (SERPINA3). Three spots of SERPINA3 were unchanged in aging, and also in this case a higher degree of S-nitrosation was observed in A but not in C (Figure 3).

**Figure 4:**
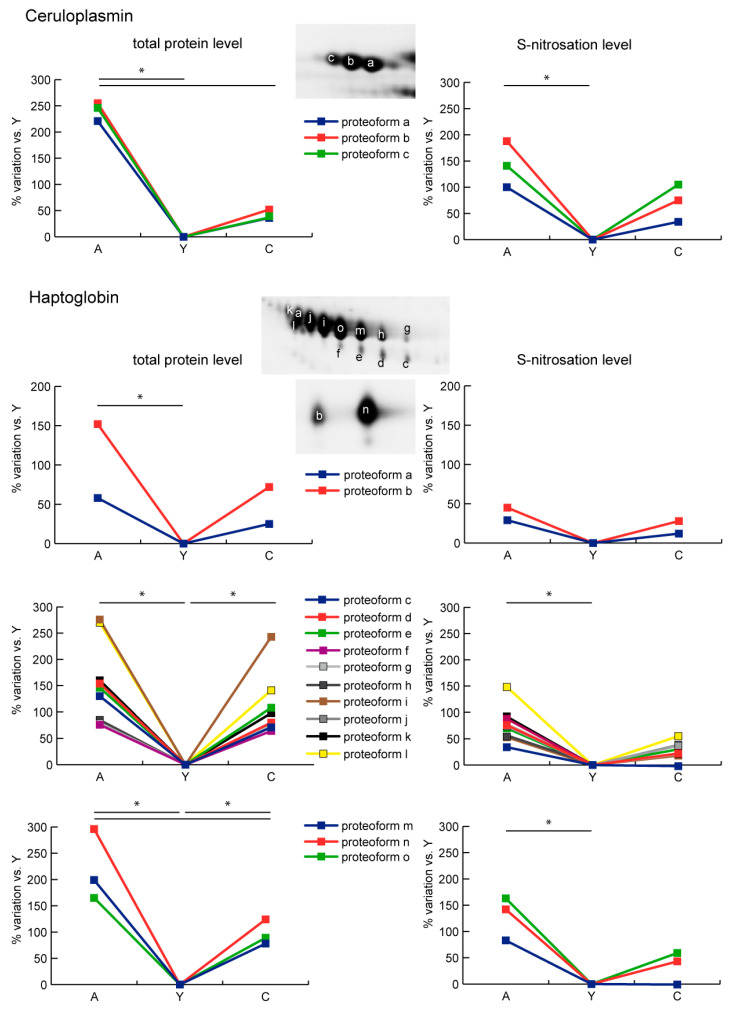
Line charts illustrating ceruloplasmin (CP) and haptoglobin (HP) proteoform abundance and S-nitrosation level variations (%) in sera of aged (A) and centenarian (C) compared to young (Y) women. Proteins were divided according to the statistical significance of the test (* = significant difference, ANOVA and Tukey, *n* = 6 (2-D DIGE) or *n* = 9 (Nitro-DIGE), *p*-value < 0.01).

As regards ceruloplasmin (CP), all the identified proteoforms were more abundant and nitrosated in A compared to Y and C. Haptoglobin (HP) is characterized by the presence of 15 proteoforms. Among them, two spots were increased in A not in C, whereas 13 increased both in A and C compared to Y. Of note, three were also significantly increased in A vs. C. Moreover, in this case, S-nitrosation appeared unchanged in proteoforms with increased protein levels in A compared to Y and C. Conversely, spot S-nitrosation of decreased proteoforms increased in A and C compared to Y, but particularly in A (Figure 4).

**Figure 5:**
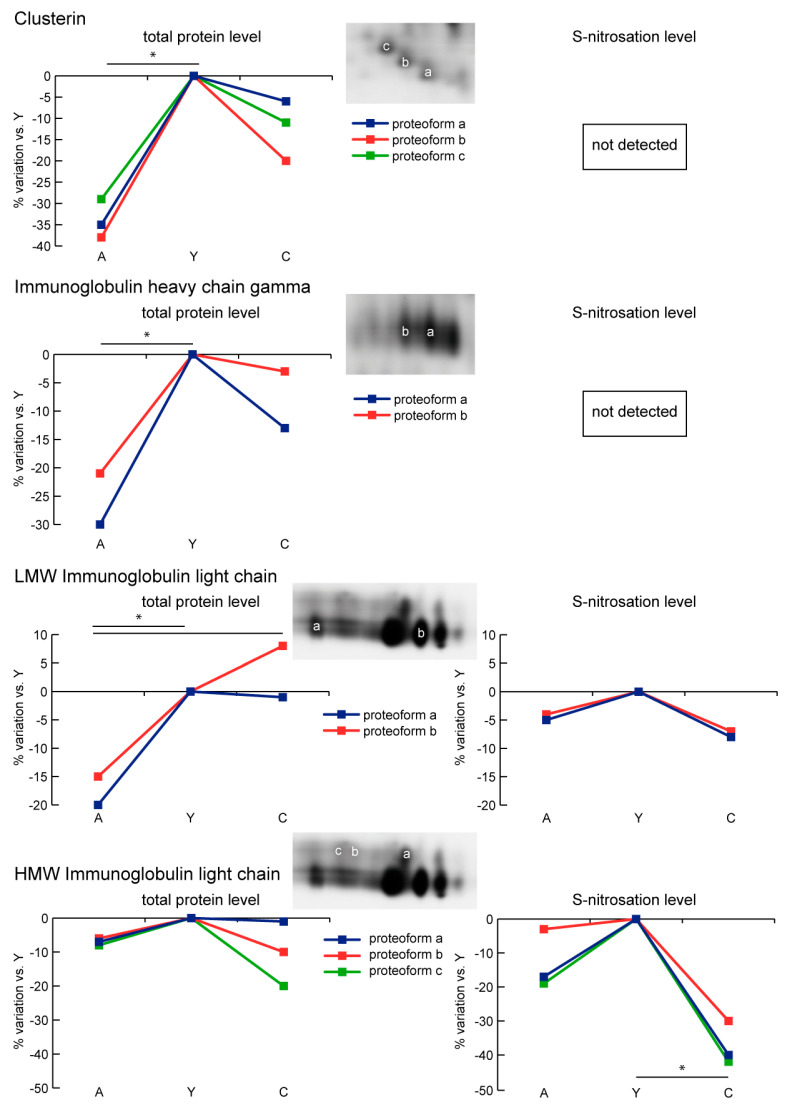
Line charts illustrating clusterin (CLU), immunoglobulin heavy chain gamma (IGHG3), and immunoglobulin light chain (IGLC1) proteoform abundance and S-nitrosation level variations (%) in sera of aged (A) and centenarian (C) compared to young (Y) women. Proteins were divided according to the statistical significance of the test (* = significant difference, ANOVA and Tukey, *n* = 6 (2-D DIGE) or *n* = 9 (Nitro-DIGE), *p*-value < 0.01).

Clusterin (CLU) and immunoglobulin heavy chain gamma (IGHG3) decreased in A vs. Y and C, and were not differentially S-nitrosated. Likewise, low molecular weight proteoforms (LMW) of immunoglobulin light chain 1 (IGLC1) decreased in A compared to Y and C. Nitrosation levels did not change significantly with age. Conversely, upper proteoforms (HMW), despite unchanged levels of total protein, showed decreased levels of S-nitrosation in C (Figure 5).

**Figure 6:**
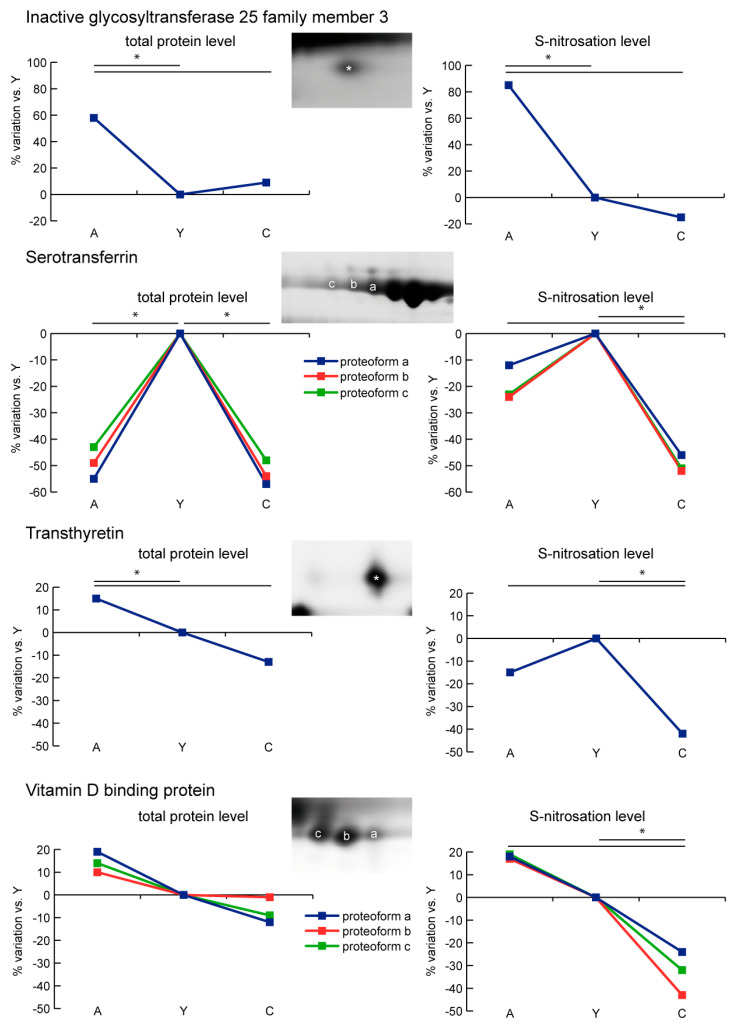
Line charts illustrating inactive glycosyltransferase 25 family member 3 (CERCAM), serotransferrin (TF), transthyretin (TTR), and vitamin D-binding protein (VDBP) proteoform abundance and S-nitrosation level variations (%) in sera of aged (A) and centenarian (C) compared to young (Y) women. Proteins were divided according to the statistical significance of the test (* = significant difference, ANOVA and Tukey, *n* = 6 (2-D DIGE) or *n* = 9 (Nitro-DIGE), *p*-value < 0.01).

Inactive glycosyltransferase 25 family member 3 (CERCAM) showed increased levels of total protein, whereas S-nitrosated CERCAM was observed in A only. Three proteoforms of serotransferrin (TF) decreased in A and C vs. Y; however, their nitrosation levels were decreased in C vs. Y only. Transthyretin (TTR) showed increased levels of total protein in A compared to Y and C. Furthermore, S-nitrosation levels decreased in C compared to Y and A. The three proteoforms of vitamin D-binding protein (VDBP) were unchanged with age; however, S-nitrosation level was lower in C in comparison to Y and A (Figure 6).

**Figure 7:**
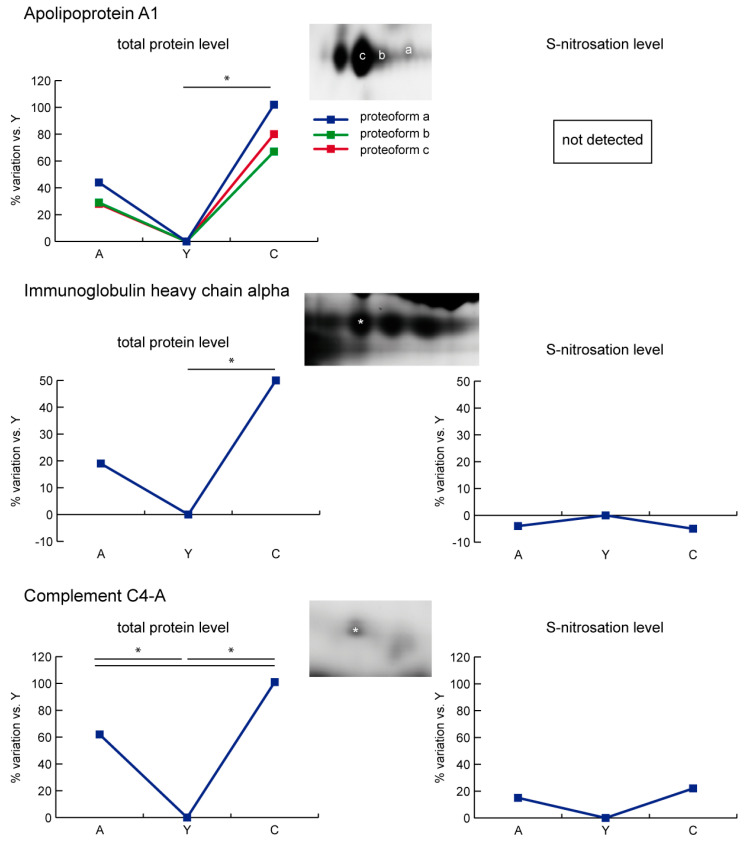
Line charts illustrating apolipoprotein A1 (APOA1), immunoglobulin heavy chain alpha (IGHA1), and complement C4-A (C4A) proteoform abundance and S-nitrosation level variations (%) in sera of aged (A) and centenarian (C) compared to young (Y) women. Proteins were divided according to the statistical significance of the test (* = significant difference, ANOVA and Tukey, *n* = 6 (2-D DIGE) or *n* = 9 (Nitro-DIGE), *p*-value < 0.01).

Three proteoforms of apolipoprotein A1 (APOA1) and immunoglobulin heavy chain alpha (IGHA1) increased in C in comparison to Y and A. The complement C4-A (C4A) resulted in an increase both in A and C vs. Y, but increased more in C vs. A. No changes in S-nitrosation were detected (Figure 7).

Concerning nitrosated proteins, albeit few exceptions (i.e., HP proteoforms c-o, CP, CERCAM and TF), we observed two general behaviors—proteoforms changed in total protein content generally did not appear to be differentially nitrosated with age (i.e., LMW SERPINA1, a and b proteoforms of HP, CLU, IGHG3, LMW IGLC1, APOA1, IGHA1, TTR in group A, C4A), whereas proteoforms that did not significantly change in total abundance were found to have different levels of S-nitrosation in aging (i.e., HMW SERPINA1, SERPINA3, HMW IGLC1, TTR in group C, VDBP).

In sum, protein S-nitrosation levels were found to be increased in A vs. Y and C, such as in HMW proteoforms of SERPINA1, SERPINA3, CP, 13 proteoforms of HP, and CERCAM. Conversely, nitrosation levels decreased in C vs. Y and A in HMW proteoforms of IGLC1, TF, TTR, and VDBP.

### 2.3. Tyrosine Nitration Assessment

Nitrated tyrosines were detected by immunoblotting. A slight increment of nitrated proteins was observed in A compared to Y and C, although this was not supported by statistical analysis (Figure S2).

### 2.4. Levels of Molecules Controlling the Nitrosative Stress in A, C, and Y Subjects

**Figure 8:**
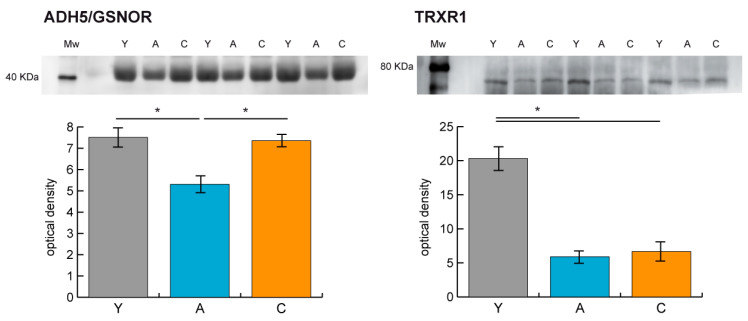
Representative histograms and immunoblot images of alcohol dehydrogenase 5/S-nitrosoglutathione reductase (ADH5/GSNOR) and thioredoxin reductase 1 (TRXR1) (mean ± SD; * = significant difference, ANOVA and Tukey’s test, *n* = 3, *p*-value < 0.05) in aged (A), young (Y), and centenarian (C) samples.

ADH5/GSNOR and thioredoxin reductase 1 (TRXR1) were assessed by immunoblotting. Figure 8 indicates that aged subjects displayed significantly lower levels of ADH5 compared to both Y and C, whereas TRXR1 levels were decreased in serum of A and C in comparison to Y.

## 3. Discussion

This pilot study was based on the serum proteome analysis of aged women in comparison with centenarian women. Results of physical performances were restricted to SPPB and hand grip test for aged women, and hand grip test for centenarians. It should be taken into account that the physical evaluation of centenarians was hampered by the unavailability of validated tools for this segment of the population, and often such procedures do not take into account the peculiar characteristics of centenarians (e.g., fatigue and sensory impairment). Nevertheless, the experience of centenarians is crucial for understanding mechanisms regulating aging and age-related conditions [31]. Sarcopenia initiates around the fourth decade of life; evidence suggests that skeletal muscle mass and skeletal muscle strength decline progressively, more in men than women, with up to 50% of mass being lost by the eighth decade of life [32,33]. Collectively, our data indicated that the decline of muscle performance was present in aged subjects, and centenarians under 105 years of age were characterized by a slower decline of muscle force and strength that became severe in very senescent subjects (106, 107, and 113 years old), where it was also difficult to perform measurements. It must also be highlighted that the majority of subjects had hypertension (12 out of 15 aged women and 11 out of 15 centenarians); however, all of them were pharmacologically treated. We were conscious about the contribution of hypertension in triggering cardiovascular disease and chronic kidney disorders (CKD); thus, due to the restricted number of subjects, particularly centenarian subjects, we decided to consider hypertension under control sharing non-hypertensive subjects homogeneously to balance their contribution inside each group.

Taking into account these preliminary observations, we based our investigation on the differential abundance of intact serum proteins and on their nitration status. The study adopted two gel-based complementary techniques (2-D DIGE and Nitro-DIGE) and unravelled specific proteoforms influenced by aging, providing hints on their level of nitrosation and describing, for the first time, levels of nitrated plasma proteins in aged subjects and of enzymes involved in the denitrosylation processes that were at variance between A and C in comparison to Y.

Overall, results indicate that for a group of proteins, nitrosation increased in A compared to Y, and, importantly, that C were characterized by lower levels of nitrosation in comparison to aged subjects, and in some cases their levels were also lower than Y.

Our data indicate that some proteins increased and had an unchanged degree of nitrosation compared to Y and C, also indicating that acidic proteoforms were preferential targets of nitrosation. Conversely, proteins with decreased levels in aging showed an increased nitrosation, and their nitrosation state was at a variance among A and C vs. Y, suggesting that this process can be associated with unbalanced homeostasis of the redox system and that these molecules can be selected as potential markers of longevity. Another underlined point is the level of SERPINA1. LMW proteoforms increased in aged subjects in comparison to Y, whereas HMW proteoforms were more nitrosated in A compared to C and Y. It could be of note that this molecule accumulates in muscle exposed to prolonged microgravity, suggesting a link between levels of this molecule in muscle tissue and its release into the bloodstream that is associated with muscle decline. Further study will be required to better clarify this issue.

Why are proteoforms with lower isoelectric point (pI) preferentially nitrosated? It can be postulated that the lower pI of proteoforms can be related to the location of the cysteine residues in the protein sequence that act as preferential targets of the sulfhydryl group for nitrosation due to their increased nucleophilicity. Thus, protein nitration is a specific process targeting only a restricted number of proteins and limited to key cysteine residues within these proteins [34].

Recent relevant studies observed that most age-associated proteins showed increased abundance with age, with the reason potentially being due to a loss of renal function in aging or a bias of the aptamer technology occurring on the basis of proteins that are targeted by the slow off-rate modified aptamers (SOMAmers) utilized to investigate the proteome [19,20,21,22,35,36]. Other proteomic studies in aged subjects using technologies such as two-dimensional gel electrophoresis [37,38] or quantitative mass spectrometry [35,39,40] showed a number of age-associated proteins that decreased as well as increased with age. In our study, the 2-D DIGE indicated more or less the same ratio of up- and downregulated, proteoforms in agreement with previous studies based on intact protein labelling and LC–MS/MS approaches. In our study, we observed increment of SERPINA1, CP, HP, CERCAM, and TTR, with some of them having already been described [18]. Furthermore, the study also indicates that specific proteoforms with lower pI were nitrosated. At variance, we observed a decrement of CLU, IGHG3, and LMW IGLC1 in A in comparison to Y and C. Among these proteins, CLU is known to enhance amyloid clearance and modulate neuroinflammation in mild cognitive impairment [41]. Its decrement can be associated with the loss of protective mechanisms in A.

Unexpectedly, proteins characterized by altered abundance in aging were also characterized by unchanged nitrosation levels and nitrosation-targeted specific proteoforms. The proteoform is a protein product of a single gene and it is a result of alternative splicing or post-translational modifications that affect proteolytic cleavage or terminal degradation [42]. Proteoforms may be depleted or enriched as they are released into the circulation. In light of this, the fact that specific plasma proteoforms are nitrosated could have implications on the origin of circulating proteins and/or in the level of NO and of molecules controlling nitrosation. The measurement of proteoforms in blood is a difficult task [43] that could in principle be overcome by mass spectrometry-based technologies, such as selected reaction monitoring (SRM) and multiple reaction monitoring (MRM); however, a strong effort on this direction is needed for it to be routinely implemented.

It has been recently described that higher levels of the denitrosylase ADH5/GSNOR enzymes promote longevity [12,44,45]. From the present study, it can be expected that the denitrosylase activity will decrease with age, as appears from the serum nitrosoprofile of A vs. Y. Results from immunoblotting indicate that a precise mechanism was targeted and that it was associated with ADH5/GSNOR, while the nitro/denitro processes under TRXR1 control were decreased both in A and C in comparison to Y.

The decrement of ADH5 confirmed our results and indicated that increased levels of nitrosated proteins both in cysteine and tyrosine (although in this case not statistically significant) in A can be due to the inhibition of ADH5/GSNOR denitrosylase activity, with C subjects appearing to be more protected from this negative signaling, sustaining the assumption that being able to denitrosylate well leads to living longer [12,13,26]. Interestingly, the level of the Trx/TrxR system was decreased both in A and C in comparison to Y, suggesting that denitrosylation in aging requires a tight regulation of the ADH5/GSNOR system while the general mechanism protecting cells from oxidative and nitrosative stress and regulating the dithiol/disulfide balance are not involved in the prolonged healthy aging status typical of centenarians.

It should be of note that specific proteins showed lower level of nitrosation in C vs. A, and they can be preferential targets of ADH5/GSNOR and therefore selected as possible targets for a precise therapeutic intervention to counteract frailty and promote healthy aging. Specifically, the HMW proteoforms of SERPINA1, SERPINA3, CP, 13 proteoforms of HP, and CERCAM being involved in tissues homeostasis can play a role on the fine-tuning of redox balance. Serpins regulate a wide range of biological processes, including inflammatory responses, and are key factors in the pathophysiology of cardiovascular diseases [46]. It could be of note that the increase of SERPINA1 and SERPINA3 in muscle and in serum was also demonstrated in various murine models of muscle atrophy [47,48], suggesting that a higher nitrosation state of these molecules could represent a trait of muscle decline. Furthermore, SERPINA3 has also been identified as a specific biomarker of delirium and Alzheimer’s disease [49,50]. CP is an acute phase reactant, regulating NO homeostasis and working as a NO oxidase, suggesting that low levels may promote NO bioavailability, protecting C from endovascular dysfunctions [51]. Another protein of interest is HP, which has been recognized as an inflammatory indicator in cardiovascular disease. It is an acute-phase protein with a role in the neutralization and clearance of free heme [51].

Of note, a set of proteins also appeared less nitrosated in comparison to younger women (i.e., IGLC1, TF, TTR, VDBP). Of major interest are the lower levels of nitrosated IGLC1. These immunoglobulin light chains contribute to antigen recognition and accumulate in chronic kidney disorders (CKDs), where their concentration progressively increases according to CKD stage, leading to tubule-interstitial lesions. IGLC1, by interfering with essential functions and apoptotic cell death of neutrophils, may contribute to infectious and inflammatory complications, which are common in CKD patients [52]. It could be speculated that constitutive lower nitration levels could be protective for renal function, which represent a hallmark of the aging functional decline. Concerning VDBP, the free hormone hypothesis postulates that only hormones bound to a binding protein are released and enter in cells, suggesting that the lower nitration state of VDBP in centenarians may promote the binding of vitamin D as a protective factor for bone formation and neuromuscular function [53,54]. Regarding TTR, it is a precursor protein of senile systemic amyloidosis and S-nitrosated transthyretins, exhibiting higher amyloidogenicity than unmodified transthyretins. Nitrosated TTR was less abundant in C, suggesting a marked ability of C to retain the healthy status [55,56]. Further specific investigations should be addressed to precisely contextualize these proteins in aging, and therein the scope of this pilot study was to provide hints for studies on this direction.

In summary, we can conclude that the proteins indicated above can be putative markers of longevity, and, in particular, they are involved in the protection of cardiovascular and renal function decline and, indirectly, of muscle mass and function decline. Furthermore, the maintenance of high-level ADH5/GSNOR can sustain denitrosylase activity, promoting longevity. A number of limitations of this study exist, since we analyzed a restricted number of samples, even though the adoption of sub-pooling allowed us to reduce the variance among biological groups, increasing the power of the study to detect changes despite the restricted number of samples [57,58]. In comparison with recent larger studies based on differential protein abundance, this study utilized complex and labor-intensive methodologies not currently available in all labs. However, this is the first study suggesting nitrosation of circulating proteins as a possible marker to predict and monitor longevity, indicating specific molecules to be targeted to prevent frailty in aging. Therefore, it has to be considered as a starting point for more focused studies on larger cohorts.

## 4. Materials and Methods

### 4.1. Participants and Ethical Statement

Serum samples were collected from female subjects grouped according to the “age at time of sampling” into young (Y; *n* = 15, age range: 32–44 years old), aged (A; *n* = 15, 76–83 years old), and centenarian (C; *n* = 15, 105–114 years old). The general characteristics of enrolled subjects are summarized in Table S1. In particular, the functionality of the lower limbs was assessed in aged subjects using the SPPB, a group of measures that combines the results of the gait speed, chair stand, and balance tests [59]. For aged subjects and centenarians, the hand grip test by manual dynamometry was utilized to easily determine musculoskeletal function, weakness, and disability [60]. The functional status was further assessed as a measurement of the subject’s ability to perform activities of daily living (i.e., bathing, dressing, toileting, transferring, continence, and feeding). The Katz index scores subjects for independence in each of these 6 functions. A score of 6 indicates full function, 4 indicates moderate impairment, and 2 or less indicates severe functional impairment.

All subjects gave their informed consent for inclusion before they participated in the study. The study was conducted in accordance with the Declaration of Helsinki, and the protocol was approved by the Ethics Committee of the Fondazione istituto di ricovero e cura a carattere scientifico (IRCCS) Ca’ Granda Ospedale Maggiore Policlinico, Milan (Protocol identification code No. 2035, amendment 30/11/2011) and of the IRCCS Fondazione Don Carlo Gnocchi, Milan (Project identification code No. 2017-0622, amendement 04/2018).

### 4.2. Serum Albumin Depletion

Before serum albumin depletion, protein concentration was carefully assessed in all samples by a Pierce bicinchoninic acid assay (BCA) Protein Assay Kit (ThermoFisher Scientific, Rodano, Italy). Albumin depletion was performed according to the manufacturer’s instructions using the Pierce Albumin Depletion Kit (ThermoFisher Scientific). Briefly, 400 µL of Pierce depletion resin (corresponding to 200 µL of settled resin) was transferred to a spin column, and the column was centrifuged and washed with 200 µL of binding/wash solution. After centrifuging at 12,000× *g* for 1 min, 50 µL of serum sample was added to the tube. The tube was incubated for 2 min at room temperature and centrifuged at 12,000× *g* for 1 min. To release unbound proteins, we washed spin tubes 3 times with 50 µL of binding/wash solution and then centrifuged them at 12,000× *g* for 1 min.

### 4.3. Sample Preparation

Serum protein concentration was quantified by BCA protein assay (ThermoFisher Scientific), and, for each group, samples were randomly selected and pooled into 3 sub-pools (5 subjects each). Proteins were selectively precipitated by acetone and resuspended in 7 M urea, 2 M thiourea, 4% 3-[(3-cholamidopropyl)dimethyl-ammonio]-1-propane sulfonate (CHAPS), 30 mM Tris, 1 mM phenylmethanesulfonyl fluoride (PMSF) (pH 8.5) (lysis buffer) for 2-D DIGE or in 300 mM sucrose, 250 mM 2-[4-(2-hydroxyethyl)-1-piperazinyl]-ethanesulfonic acid (HEPES) (pH 7.7), 1 mM Ethylenediaminetetraacetic acid (EDTA), and 0.1 mM neocuproine (HEN buffer) for Nitro-DIGE. After resuspension, protein concentration of sub-pools was determined by 2-D quant Kit (GE Healthcare) before 2-D DIGE. For Nitro-DIGE, sample preparation was carried out in the dark to prevent SNO decomposition. Protein concentration was determined using the BCA Protein Assay Kit and adjusted to 1 mg/mL with HEN buffer.

### 4.4. Two-Dimensional Difference in Gel Electrophoresis (2-D DIGE)

2-D DIGE was conducted on albumin-depleted serum extracts from 9 different sub-pools, each one constituted of 5 different age- and sex-matched subjects. All experimental procedures such as protein labeling, 2-D separation parameters, and protein analyses were performed as previously described [61]. For protein minimal labeling, cyanine dyes (Cy3 and Cy5) were adopted according to the manufacturer’s instructions (GE healthcare, Little Chalfont, Buckinghamshire, UK). Briefly, 50 µg of each sub-pool extract was mixed with 400 pmol of CyDye (Cy5); the reaction was performed on ice in the dark for 30 min. The reaction was blocked by adding 1 mL L-lysine 10 mM for 10 min on ice. Internal standard (Cy3-labelled) was prepared by mixing 100 µg of each sub-pool (Y, A, and C). After protein labeling, 40 µg of each sample and 40 µg of internal standard were mixed. Samples were run in duplicate using 24 cm, 3–10 non-linear pH-gradient immobilized pH gradient (IPG) strips. Isoelectric focusing was performed on an IPGphor electrophoresis unit (GE Healthcare) using a gradient ranging from 200 to 8000 V, reaching a total of 75,000 Vh. Focused proteins on IPG strips were prepared for second dimension by reduction and alkylation. Second dimension was carried out using the Ettan Dalt II system (GE Healthcare) on 20 × 25 cm^2^, 12% T, 2.5% C constant concentration polyacrylamide gels at 20 °C and 15 mA.

### 4.5. Identification of S-Nitrosated Proteins by 2-D CyDye-Maleimide DIGE (Nitro-DIGE)

A modified biotin switch method [62] with CyDye maleimide monoreactive sulfhydryl-reactive fluorescent dyes (GE Healthcare) to identify SNO proteins was used. Free thiols were blocked with 4X volume of 50 mM iodoacetamide (IAA) in HEN buffer containing 2.5% SDS for 30 min. Excess IAA was removed by cold acetone precipitation. Protein pellets were washed; dissolved in HEN buffer containing 1% SDS, 5 mM sodium ascorbate, and 1 µM copper sulfate [63]; and incubated at room temperature for 1 h in order to reduce S-nitrosothiols. After acetone precipitation, proteins were dissolved in labeling buffer (30 mM Tris-HCl (pH 8), 8 M urea, 4% CHAPS) at 2.5 mg/mL. CyDye DIGE Fluor reagent (10 μM Cy3 or Cy5) was added to each sample and incubated at room temperature for 1 h to label NO-released thiols. Each group consisted of at least three biological replicates; each replicate was labeled with Cy5, and a mixture containing an equal amount of all samples was labeled with Cy3 as the internal standard. After quenching with 50 mM dithiothreitol (DTT), labeled samples (internal standard versus each replicate) were mixed and separated by two-dimensional electrophoresis, as previously described.

### 4.6. Image Acquisition and Statistical Analysis

Images from CyDye-labeled gels were acquired by Typhoon 9200 Imager (GE Healthcare), and image analysis was performed by DeCyder software (version 6.5, GE Healthcare). Three groups of subjects were analyzed, and for each group (Y, A, and C), we only considered spots present in at least 90% of the samples. 2-D DIGE and Nitro-DIGE statistically significant differences were computed by analysis of variance (ANOVA) and corrected for Tukey’s test (*p*-value < 0.01); when the use of ANOVA was not possible, the non-parametric Kruskal–Wallis (*p*-value < 0.01) test was adopted. False discovery rate was applied to correct for multiple tests to reduce the overall error. Statistically changed proteins underwent the power analysis, and only spots reaching a sensitivity cut-off > 0.8 were considered as differentially expressed.

### 4.7. Protein Identification

Protein identification was carried out by comparison of gel images to a human plasma reference 2-D map publicly available (https://world-2dpage.expasy.org/swiss-2dpage/viewer). Proteins not annotated in this map were identified by matrix-assisted laser desorption/ionization time-of-flight (MALDI-TOF) mass spectrometry (MS). For protein identification, semi-preparative gels were loaded with unlabelled sample (400 μg per strip); electrophoretic conditions were the same as 2-D DIGE, and gels were stained with a total-protein fluorescent stain (Krypton, ThermoFisher Scientific). Image acquisition was performed using a Typhoon 9200 laser scanner. Spots of interest were excised from gel using the Ettan spot picker robotic system (GE Healthcare), destained in 50% methanol/50 mM ammonium bicarbonate, and incubated with 30 μL of 6 ng/mL trypsin (Promega, Madison, Wisconsin, USA) dissolved in 10 mM ammonium bicarbonate for 16 h at 37 °C. Released peptides were subjected to reverse-phase chromatography (Zip-Tip C18 micro, Merck Millipore, Milano, Italy), eluted with 50% acetonitrile (ACN)/0.1% trifluoroacetic acid. Peptide mixture (1 μL) was diluted in an equal volume of 10 mg/mL alpha-cyano-4- hydroxycinnamic acid matrix dissolved in 70% ACN/30% citric acid and processed on an Ultraflex III MALDI-TOF/TOF (Bruker Daltonics, Bremen, Germany) mass spectrometer. MS was performed at an accelerating voltage of 20 kV, and spectra were externally calibrated using Peptide Mix calibration mixture (Bruker Daltonics); 1000 laser shots were taken per spectrum. Spectra were processed by FlexAnalysis software v. 3.0 (Bruker Daltonics), setting the signal to noise threshold value to 6, and search was carried out by correlation of uninterpreted spectra to *Homo sapiens* entries in Uniprot Proteomes UP5640 20200812 (97,065 sequences; 38,762,114 residues) using BioTools v. 3.2 (Bruker Daltonics) interfaced to the on-line MASCOT software (Matrix Science, London, UK) which utilizes a robust probabilistic scoring algorithm. The significance threshold was set at a *p*-value < 0.05. No mass and pI constraints were applied, and trypsin was set as enzyme. One missed cleavage per peptide was allowed, and carbamidomethylation was set as fixed modification while methionine oxidation was set as variable modification. Mass tolerance was set at 30 ppm for MS spectra.

In cases where this approach was unsuccessful, we performed additional searches using electrospray ionization–MS/MS, as previously described [64].

### 4.8. Immunoblotting

Protein extracts (50 μg) from pooled Y, A, and C serum samples were loaded in triplicate and resolved on 12–18% gradient polyacrylamide gels. Blots were incubated with rabbit or mouse primary antibodies as follows: anti-nitrotyrosine (Cayman Chemicals, Ann Arbor, Michigan, USA, no. 10189540, 1:200), anti-ADH5 (Santa Cruz Biotechnology, Dallas, Texas, USA, sc-293460, 1:500), anti-TRXR1 (Novus Biologicals, Bio-Techne, Milano, Italy, NBP1-81791, 1:500). After washing, membranes were incubated with anti-rabbit (GE Healthcare, 1:10,000) or anti-mouse (KPL, Seracare, Milford, Massachusetts, USA, 1:5000) secondary antibodies conjugated with horseradish peroxidase. Signals were visualized by chemiluminescence using the ECL Prime Detection Kit and the Image Quant LAS 4000 (GE Healthcare) analysis system. Band quantification was performed using the Image Quant TL v. 8.1(GE Healthcare) software followed by statistical analysis (ANOVA + Tukey, *n* = 3, *p*-value < 0.05). Band intensities were normalized against the total amount of proteins stained by Sypro ruby total-protein stain.

## Supplementary Materials

The following are available online at https://www.mdpi.com/1422-0067/21/23/9009/s1.

## Abbreviations

2-D DIGE	Two-dimensional difference in gel electrophoresis
ADH5/GSNOR	Alcohol dehydrogenase 5/S-nitrosoglutathione reductase
ANOVA	Analysis of variance
APOA1	Apolipoprotein A1
BCA	Bicinchoninic acid assay
BMI	Body mass index
C4A	Complement C4-A
CERCAM	Inactive glycosyltransferase 25 family member 3
CHAPS	3-[(3-cholamidopropyl)dimethyl-ammonio]-1-propane sulfonate
CKD	Chronic kidney disorder
CLU	Clusterin
CP	Ceruloplasmin
DTT	Dithiothreitol
EDTA	Ethylenediaminetetraacetic acid
GSNO	S-nitrosoglutathione
HEPES	2-[4-(2-hydroxyethyl)-1-piperazinyl]-ethanesulfonic acid
HMW	High molecular weight
HP	Haptoglobin
IAA	Iodoacetamide
IGHA1	Immunoglobulin heavy chain alpha
IGHG3	Immunoglobulin heavy chain gamma
IGLC1	Immunoglobulin light chain 1
IPG	Immobilized pH gradient
IRCCS	Istituto di ricovero e cura a carattere scientifico (scientific institute for research, hospitalization and healthcare)
LC–MS/MS	Liquid chromatography tandem mass spectrometry
LMW	Low molecular weight
MALDI-TOF	Matrix-assisted laser desorption/ionization time-of-flight
MRM	Multiple reaction monitoring
MS	Mass spectrometry
NADPH	Nicotinamide adenine dinucleotide phosphate, reduced form
Nitro-DIGE	2-D CyDye-maleimide difference in gel electrophoresis
NO	Nitric oxide
NOS	NO synthase
PBMC	Peripheral blood mononuclear cells
pI	Isoelectric point
PMSF	Phenylmethanesulfonyl fluoride
RNS	Reactive nitrogen species
ROS	Reactive oxygen species
RYR1	Ryanodine receptor 1 (skeletal muscle type)
RYR2	Ryanodine receptor 2 (cardiac muscle type)
SERPINA1	Alpha-1-antitripsin
SERPINA3	Alpha-1-antichimotripsin
SNOs	Nitroso thiols
SOMAmers	Slow off-rate modified aptamers
SPPB	Short physical performance battery
SRM	Selected reaction monitoring
TF	Serotransferrin
Trx/TrxR	Thioredoxin/thoredoxin reductase
TRXR1	Thioredoxin reductase 1
TTR	Transthyretin
VDBP	Vitamin D-binding protein
