# Supplementary material for: Can Serum Nitrosoproteome Predict Longevity of Aged Women?
Source: Int J Mol Sci. 2020 Nov 27;21(23):9009. doi: 10.3390/ijms21239009 (PMC7731247; doi:10.3390/ijms21239009)
Supplement: Supplementary file 1 [file ijms-21-09009-s001.zip › ijms-994736-2refinal-supplementary/Supplementary Figures S1 and S2.docx]

**
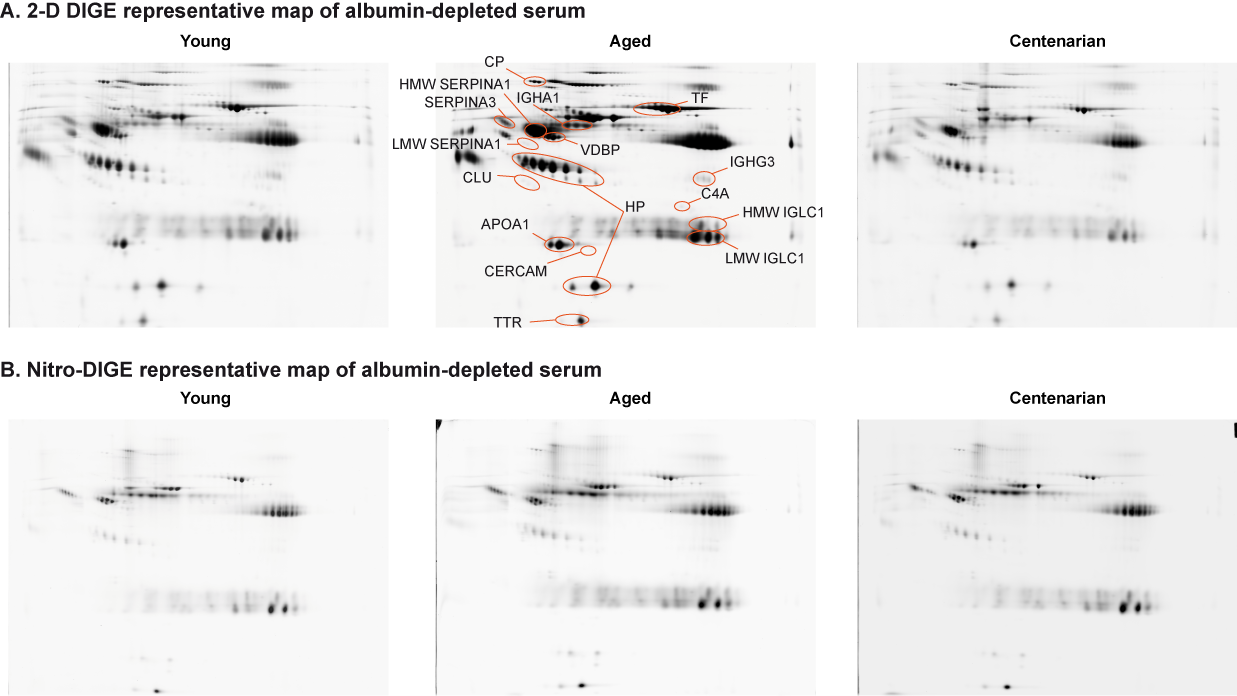
**

**Supplementary Figure S1.** Representative 2-D maps showing: **(A)** total protein content of albumin-depleted serum obtained using 2-D DIGE; **(B)** serum S-nitrosoproteome obtained from Nitro-DIGE referred to different experimental conditions (Young, Aged and Centenarian). Protein identification was carried out by comparison of gel images to a human plasma reference 2-D map publicly available (https://world-2dpage.expasy.org/swiss-2dpage/viewer). Proteins not annotated in the reference map were identified by matrix-assisted laser desorption/ionization–time-of-flight (MALDI-ToF) mass spectrometry (MS).


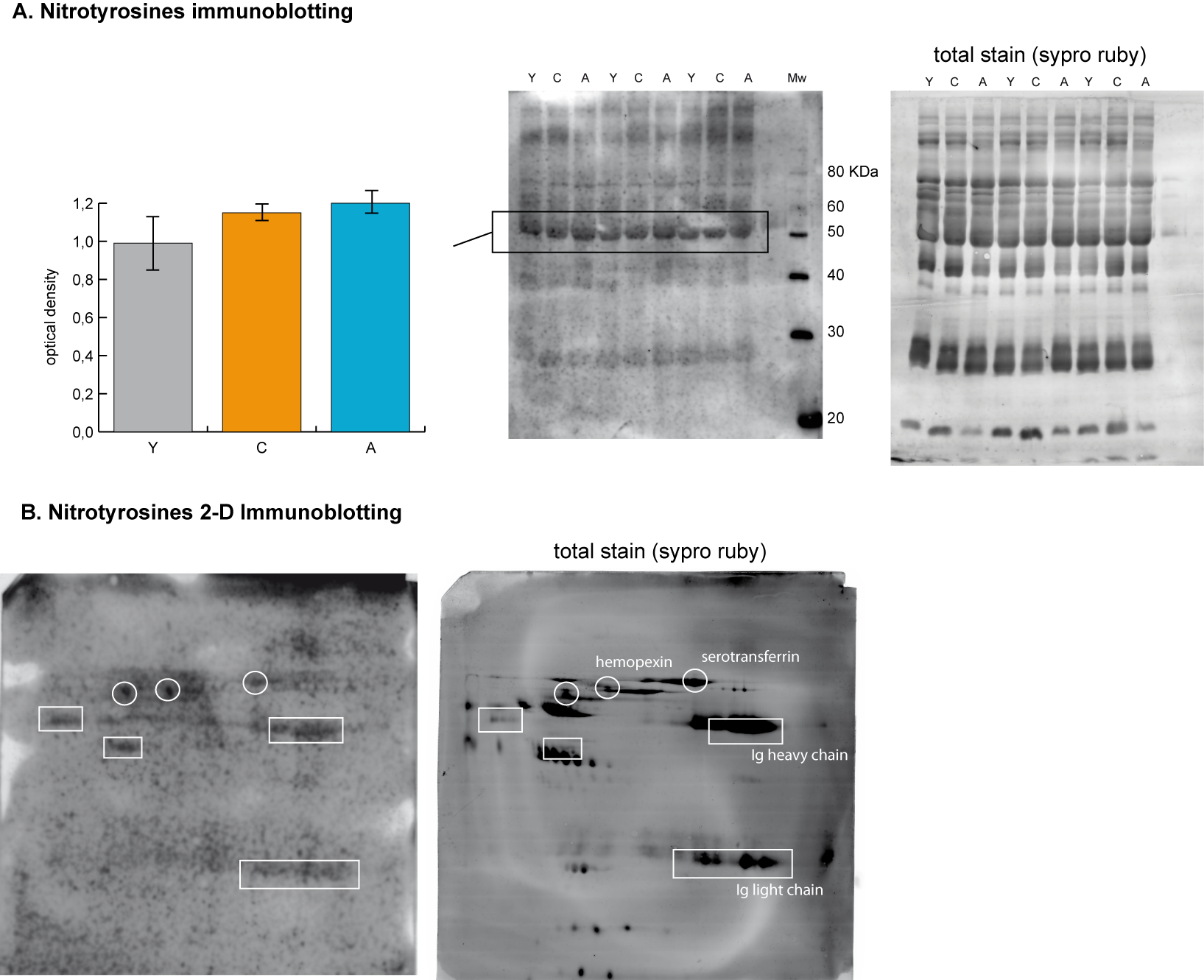


**Supplementary figure S2.** **(A)** Representative histograms and immunoblot images of proteins containing nitrated tyrosines (mean ± SD; ANOVA and Tukey’s test, n = 3, P-value < 0.05) in aged (A), young (Y) and centenarian (C) samples. Band intensities were normalized against the total amount of proteins stained by Sypro ruby total-protein stain as loading control. A slight increment of nitrated proteins was observed in A compared to Y and C, although not supported by statistical analysis. **(B)** Representative 2-D immunoblot image of nitrotyrosines in aged pool (left) compared to the total protein content (right). 2-D immunoblots were performed by focusing albumin depleted sera on 13 cm, 3-10 pH gradient IPG strips prior to SDS-PAGE separation on 12% polyacrylamide gels. Identification of nitrated proteins was carried out by comparison of gel images to a human plasma reference 2-D map publicly available (https://world-2dpage.expasy.org/swiss-2dpage/viewer).
